# Supplementary material for: Three-Dimensional Microfibrous Scaffold with Aligned Topography Produced via a Combination of Melt-Extrusion Additive Manufacturing and Porogen Leaching for In Vitro Skeletal Muscle Modeling
Source: Bioengineering (Basel). 2024 Mar 28;11(4):332. doi: 10.3390/bioengineering11040332 (PMC11047940; doi:10.3390/bioengineering11040332)
Supplement: Supplementary file 1 [file bioengineering-11-00332-s001.zip › bioengineering-2827001-supplementary.pdf]

bioengineering

MDPI

Article

3D micro-fibrous scaffold with aligned topography produced via a combination of Melt-Extrusion Additive Manufacturing and porogen leaching for *in vitro* skeletal muscle modelling

Mattia SPEDICATI [1,2,3], Alice ZOSO[1,2,3], Leonardo MORTATI[4], Valeria CHIONO[1,2,3], Elena MARCELLO[1,2,3*] and Irene CARMAGNOLA[1,2,3*]

[1] Department of Mechanical and Aerospace Engineering, Politecnico di Torino, Torino, Italy;
[2] POLITO BioMedLab, Politecnico di Torino, Torino, Italy;
[3] Interuniversity Center for the promotion of the 3Rs principles in teaching and research, Italy;
[4] Istituto Nazionale di Ricerca Metrologica (INRIM), Torino, Italy.
* These authors share last authorship. Correspondence: elena.marcello@polito.it; irene.carmagnola@polito.it;

Supplementary

S.1. Thermal characterization of polimeric blend

Table S1. Thermal properties of PCL and PEG polymers and their blends as determined through DSC analysis. Tm is the melting peak and ΔHm is the enathalphy of fusion in mJ/mg.

	Melting peak (Tm) °C	Range of melting °C	Enthalpy of fusion (ΔHm) mJ/mg
**PCL**	65	30-75	85
**PEG**	66	40-80	177
**60/40**	69	40-80	139
**50/50**	68	40-80	135
**40/60**	68.5	40-80	140

S.2. PCL outer shell formation and printing parameter optimization

To evaluate the influence of printing speed and pressure on the fiber quality, preliminary work was conducted using 50/50_A scaffold, 7.5 mm/s of printing speed and 40 kPa. These parameters led to the formation of a PCL outer shell formation as shown in Figure S1 (a). As shown in SEM images (Figure S1 (b) and (c)) of a filament section fractured in liquid nitrogen, this thin PCL shell surrounded the inner PCL fibers that were formed after the porogen leaching process, resulting in an uneven and non-fibrous scaffold (Figure S1 (a)). Additionally, the presence of the PCL shell remained unaffected by any residual PEG, as evidenced by the efficacy of the porogen leaching process. This was demonstrated through a weight loss test, which revealed the complete removal of the PEG fraction within a span of 3 days. Parameters were tuned to avoid shell formation and ensure the best post-leaching results. Considering previous literature [54], extrusion pressure and printing speed were lowered to reduce shear stress formation during the printing process within the melted PCL/PEG polymeric blend that allegedly caused the formation of the

outer PCL shell. Pressure was then set to 10 kPa, the lowest values that allowed a good material extrusion. Then, print speeds were increased starting from 1 mm/s and moving upwards at 0.5 mm/s steps, until the detection of the lowest speed that could allow a shear stress to align with the PCL/PEG molten polymeric phase. The final printing speed was thus set to 2 mm/s, reaching a good balance between structural integrity and fiber homogeneity, while hindering the formation of a PCL outer shell.

S.3. Porogen leaching process timing optimization

To assess the shortest porogen leaching process duration, the 50/50_A scaffolds weight was monitored during the leaching process over time, until the obtainment of 50% final weight loss, corresponding to the complete solubilization of PEG weight fraction. Dried sample weight was measured every day after water refresh, and weight loss was obtained through Equation 2 (reported in paragraph *2.4. Porogen leaching*). Results, reported in Figure S2, show that the highest mass loss occurred during the first 2 days of leaching and the weight remained stable between 2 and 3 days of water immersion. Therefore, a leaching time of three days was selected as optimal for complete porogen removal.

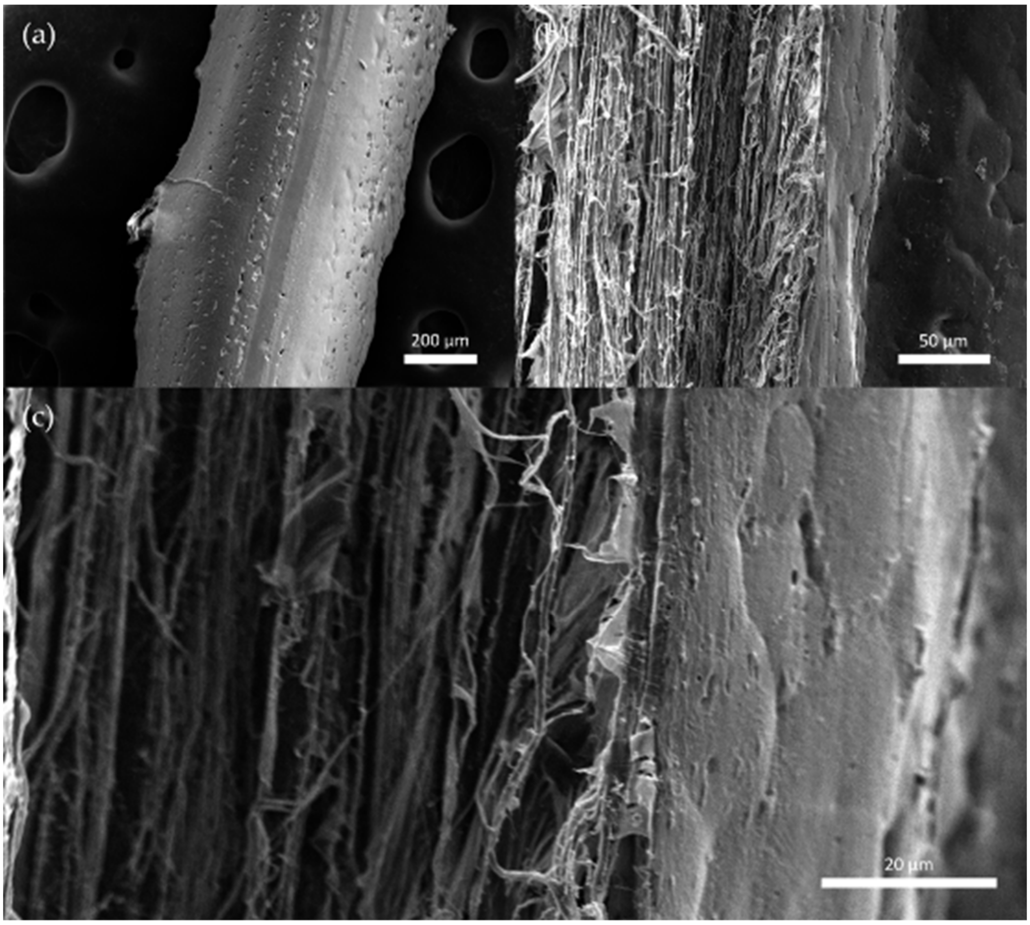

Figure S1. SEM images of 50/50_A scaffold showing the formation of PCL outer shell and fiber formation inside each filament: (a) image of filament outer surface and section of filament cut with fragile rupture with liquid nitrogen, taken at (b) 500x and at (c) 1200x.

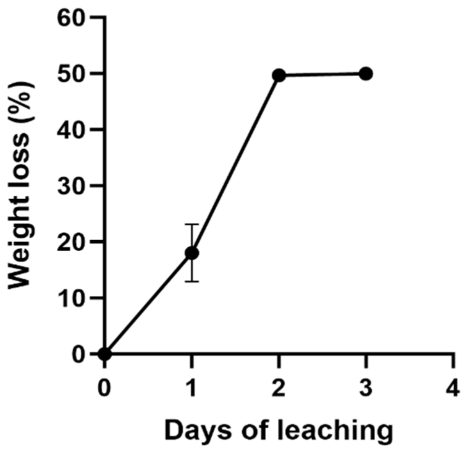

Figure S2. Curve showing weight loss percentage, due to PEG dissolution, during porogen leaching for a 50/50_A scaffold.
